# The bidirectional association of 24-h activity rhythms and sleep with depressive symptoms in middle-aged and elderly persons

**DOI:** 10.1017/S003329172100297X

**Published:** 2023-03

**Authors:** Maud de Feijter, Desana Kocevska, M. Arfan Ikram, Annemarie I. Luik

**Affiliations:** 1 Department of Epidemiology, Erasmus MC University Medical Center, Rotterdam, the Netherlands; 2 Department of Child and Adolescent Psychiatry, Erasmus MC University Medical Center, Rotterdam, the Netherlands; 3 Department of Sleep and Cognition, Netherlands Institute for Neuroscience, Amsterdam, the Netherlands

**Keywords:** 24-h activity rhythm, actigraphy, depressive symptoms, longitudinal study, middle-aged and elderly persons, population-based

## Abstract

**Background:**

In older populations disturbed 24-h activity rhythms, poor sleep, and depressive symptoms are often lingering and co-morbid, making treatment difficult. To improve insights into these commonly co-occurring problems, we assessed the bidirectional association of sleep and 24-h activity rhythms with depressive symptoms in middle-aged and elderly persons.

**Methods:**

In 1734 participants (mean age: 62.3 ± 9.3 years, 55% women) from the prospective Rotterdam Study, 24-h activity rhythms and sleep were estimated with actigraphy (mean duration: 146 ± 19.6 h), sleep quality with the Pittsburgh Sleep Quality Index, and depressive symptoms with the Center for Epidemiological Studies Depression scale. Repeated measures were available for 947 participants (54%) over a median follow-up of 6 years (interquartile range = 5.6–6.3). Linear-mixed models were used to assess temporal associations of 24-h activity rhythms and sleep with depressive symptoms in both directions.

**Results:**

High 24-h activity rhythm fragmentation (IV) (*B* = 1.002, 95% confidence interval (CI) = 0.641–1.363), long time in bed (TIB) (*B* = 0.111, 95% CI = 0.053–0.169), low sleep efficiency (SE) (*B* = −0.015, 95% CI = −0.020 to −0.009), long sleep onset latency (SOL) (*B* = 0.009, 95% CI = 0.006–0.012), and low self-rated sleep quality (*B* = 0.112, 95% CI = 0.0992–0.124) at baseline were associated with increasing depressive symptoms over time. Conversely, more depressive symptoms at baseline were associated with an increasing 24-h activity rhythm fragmentation (*B* = 0.002, 95% CI = 0.001–0.003) and TIB (*B* = 0.009, 95% CI = 0.004–0.015), and a decreasing SE (*B* = −0.140, 95% CI = −0.196 to −0.084), SOL (*B* = 0.013, 95% CI = 0.008–0.018), and self-rated sleep quality (*B* = 0.193, 95% CI = 0.171–0.215) over time.

**Conclusion:**

This study demonstrates a bidirectional association of 24-h activity rhythms, actigraphy-estimated sleep, and self-rated sleep quality with depressive symptoms over a time frame of multiple years in middle-aged and elderly persons.

## Introduction

Poor sleep and depressive symptoms are highly common (Franzen & Buysse, 2008; Murphy & Peterson, 2015), especially in middle-aged and elderly persons (Bao et al., 2017; Luik, Zuurbier, Hofman, Van Someren, & Tiemeier, 2013). Moreover, symptoms frequently co-occur (Franzen & Buysse, 2008; Vandeputte & de Weerd, 2003), and are accompanied by high physical distress and poor quality of life (Franzen & Buysse, 2008; Olfson, Wall, Liu, Morin, & Blanco, 2018). The co-occurrence of poor sleep and depressive symptoms does not merely reflect high prevalence of these disorders in old age (Fang, Tu, Sheng, & Shao, 2019). Instead, a causal link is suggested by clinical trials showing that psychological treatment of insomnia reduces depressive symptoms (Gee et al., 2018) and conversely that psychological treatment of depressive symptoms improves sleep and insomnia (Yon et al., 2014). Furthermore, population-based studies reported poor sleep to be predictive for onset and recurrence of depression (Bao et al., 2017; Hertenstein et al., 2019; Smagula et al., 2015), and depressive symptoms to be predictive for development and worsening of sleep disturbances (Bao et al., 2017; Sun et al., 2018).

There are several hypotheses about what aspect of the brain could underlie these associations as a common cause or mechanism (Asarnow, 2019; Bao et al., 2017; Beck, 2013; Brudzynski, 2014; Swaab, Fliers, & Partiman, 1985; Vadnie & McClung, 2017); for example, deterioration of the superchiasmatic nucleus (SCN), because of its prominent role in the circadian sleep-wake cycle and functioning of the hypothalamic–pituitary–adrenal (HPA) gland (Asarnow, 2019; Swaab et al., 1985). Additionally, the prefrontal cortex, because of its role cognitive functioning (Beck, 2013), and the ascending reticular activating system, because of its role in health perception (Brudzynski, 2014), have also commonly been suggested as potentially underlying the sleep–depression link. Yet, to understand underlying mechanisms, we first need to improve our understanding of the bidirectional association between sleep and depressive symptoms within the same time-span and population.

Our understanding of the bidirectional association is hampered because most studies use cross-sectional data or assess only one direction of the association. To estimate possible bidirectionality, it is required to investigate both directions of the association within a single-study population, using similar measurements and covariates. Furthermore, research has mainly looked into the effect of sleep duration (Zhai, Zhang, & Zhang, 2015), overall self-rated sleep quality (Bao et al., 2017), or insomnia (Fang et al., 2019) when taking bidirectionality into account, neglecting other aspects of sleep, such as sleep efficiency (SE) and sleep onset latency (SOL), and 24-h activity rhythms which may play an important role as well (de Feijter, Lysen, & Luik, 2020; Luik et al., 2015). Investigating multiple aspects of sleep could provide new insights important to understand the biological mechanisms underlying the association between poor sleep and depressive symptoms, and help us to develop and improve non-pharmacological and pharmacological treatments for poor sleep and depression.

We assessed the bidirectional association of 24-h activity rhythms, actigraphy-estimated sleep, and self-rated sleep quality with depressive symptoms to gain more insight into the temporality of these associations. Specifically, we estimated how 24-h activity rhythms and sleep at baseline were associated with depressive symptoms over time and its change over time. Vice versa, we also assessed how depressive symptoms at baseline were associated with 24-h activity rhythms and sleep over time and change in 24-h activity rhythms and sleep over time. Moreover, as these associations may be dependent on several factors, we assessed whether these associations were independent of a range of factors commonly associated with poor sleep and depression. To this end, we used repeatedly collected data from the prospective Rotterdam Study, a large population-based cohort of middle-aged and elderly persons.

## Methods

### Participants and design

Participants were included from the Rotterdam Study, an ongoing population-based cohort of middle-aged and elderly inhabitants of Rotterdam, the Netherlands. The study started in 1990 to investigate prevalence, history, and risk factors of common disease in elderly. So far, 14 926 participants aged ⩾45 years were included. Further details of the study design have been described elsewhere (Ikram et al., 2020).

From December 2004 until April 2007, we invited 2614 participants to wear an actigraph for 1 week for our baseline assessment, 2071 (79%) agreed. Of those that agreed, 279 participants were excluded because of incomplete or invalid baseline actigraphy (<4 complete 24-h periods of actigraphy data or <4 complete nights of actigraphy and sleep diary). Another 20 participants were excluded because of incomplete data on depressive symptoms. Finally, we excluded 38 participants because of a Mini Mental State Examination (Tombaugh & McIntyre, 1992) score below 23 or missing score as a poor cognitive status might impair the ability to complete questionnaires validly. Therefore, a total of 1734 participants were included at baseline.

Between February 2011 and July 2014, we aimed to follow-up these 1734 persons. Of those, 100 persons died, 326 withdrew from the overall study or refused to participate in the actigraphy sub-study, and 167 were not invited due to logistic reasons, leaving 1141 persons that participated in the second round of the actigraphy sub-study. For 194 participants measurements were excluded because data on 24-h activity rhythm, sleep, or depressive symptoms were incomplete or invalid at follow-up. Therefore, repeated data were available for 947 participants (55%) with a median follow-up of 6 years [interquartile range (IQR) = 5.6–6.3]. A flow diagram of the study population is presented in online Supplementary Table S1.

The Rotterdam Study has been approved by the Medical Ethics Committee of the Erasmus MC (registration number MEC 02.1015) and by the Dutch Ministry of Health, Welfare and Sport (Population Screening Act WBO, license number 1071272-159521-PG). The Rotterdam Study has been entered into the Netherlands National Trial Register (NTR; http://www.trialregister.nl/trial/6645) meeting the requirements by the WHO International Clinical Trials Registry Platform (ICTRP; http://www.who.int/ictrp/network/primary-registries/) under shared catalog number NTR6831. All participants provided written informed consent to participate in the study and to have their information obtained from treating physicians. The authors assert that all procedures contributing to this study comply with the ethical standards of the relevant national and institutional committees on human experimentation and with the Helsinki Declaration of 1975, as revised in 2008.

### Measurement of 24-h activity rhythms and sleep

All participants were asked to wear an actigraph on their non-dominant wrist for 7 consecutive days and nights, and complete a sleep diary at the same time. Additionally, participants were asked to press a marker button on the actigraph when they initiated sleep (time to bed) and when they got out of bed (get-up time). At baseline, we used the ActiWatch, model AW4 (Cambridge Technology Ltd, Cambridge, UK), and at follow-up either the ActiWatch or the GENEActiv (ActivInsight Ltd, Kimbolton, UK) was used. Recordings were sampled at 32 Hz (ActiWatch) or 50 Hz (GENEActiv) and scored for each 30-s epoch, taking into account the weighted scores of previous and following epochs. To distinguish sleep from wake a threshold of 20 was used for each 30-s epoch (Kosmadopoulos, Sargent, Darwent, Zhou, & Roach, 2014). The *z*-axis data of the tri-axial GENEActiv data were pre-analyzed to make records comparable with ActiWatch data (te Lindert & Van Someren, 2013). Actigraphy is a validated and commonly used instrument to objectively estimate sleep (Morgenthaler et al., 2007), and 24-h activity rhythms (Ancoli-Israel et al., 2003), also in population-based samples (Marino et al., 2013).

The 24-h activity rhythm was estimated by analyzing actigraphy data with the nparACT R package (Blume, Santhi, & Schabus, 2016). We calculated the interdaily stability (IS), which indicates the stability of the rhythm over days, and intradaily variability (IV), which indicates the fragmentation of the rhythm relative to its 24-h amplitude (Van Someren, 1997).

To estimate sleep, data from the actigraphy were supplemented with information from the sleep diary or marker button. Time in bed (TIB) was calculated as the difference between time to bed and get-up time. If time to bed or get-up time was missing from the sleep diary, information from the actigraphy marker button was used. Total sleep time (TST) was calculated as the total duration of epochs scored as sleep. SE was defined as the proportion of TIB spent sleeping [100% × (TST/TIB)]. SOL was defined as the time it took participants to fall asleep from time to bed. Wake after sleep onset (WASO) was calculated as the total time of the epochs scored as wake between sleep start and sleep end.

Self-rated sleep quality was determined with the widely used Pittsburgh Sleep Quality Index (PSQI), a 19-item self-report scale on sleep quality (Buysse et al., 1991). The PSQI has also been validated for measuring self-rated sleep quality in population-based samples (Mollayeva et al., 2016). The total score was calculated as the sum of all items and ranges from 0 to 21, with a high score indicating poor sleep quality. For participants with 6 out of 7 valid PSQI component scores, a weighted global score was calculated by multiplying with 7/6. If less than 6 component scores were available, PSQI was set to missing.

### Measurement of depressive symptoms

Depressive symptoms were assessed using Dutch version of the Center for Epidemiologic Studies Depression scale (CES-D), which consists of 20 items (Beekman, Van Limbeek, Deeg, Wouters, & Van Tilburg, 1994; Radloff, 1977). The CES-D has been validated for use in middle-aged and older adults (Beekman et al., 1994) and population-based cohorts (Cosco, Prina, Stubbs, & Wu, 2017). The total score ranges from 0 to 60, with a higher score indicating more severe depressive symptoms. A weighted score was calculated if ⩾75% of the questions were completed, if less than 75% of the items was completed, CES-D was set to missing.

### Other variables

Based on previous literature, the following variables were assessed as possible confounders: age, sex, employment, education, partnership, smoking behavior, coffee intake, alcohol intake, and body mass index (BMI), and general cognitive function (Luik et al., 2013; Sun et al., 2018). During the home interview information on employment, education, partnership, smoking behavior, and cognitive status was obtained. Education was classified as primary education (primary), lower/intermediate general education or lower vocational education (low), intermediate vocational education or higher general education (middle), or higher vocational education or university (high). Smoking behavior was classified as never, former, or current smoker. Intake of coffee and alcohol were assessed in the sleep diary as cups per day after 6 PM. To calculate BMI (kg/m^2^), height and weight were assessed on calibrated scales at the research center without heavy clothing and shoes.

Additionally, we determined general cognitive function by means of the *g*-factor (Deary, 2012). The *g*-factor was estimated using principal component analyses including the color-word interference subtask of the Stroop test (Houx, Jolles, & Vreeling, 1993), the Letter Digit Substitution Task (Lezak, Howieson, Loring, & Fischer, 2004), a verbal Word Fluency Test (Welsh et al., 1994), the delayed recall score of a 15-word Word List Learning test (Bleecker, Bolla-Wilson, Agnew, & Meyers, 1988), and Purdue pegboard test (Tiffin & Asher, 1948).

### Statistical analyses

Descriptives are presented as number with percentage for categorical variables and mean with standard deviation (s.d.) for numerical data. To assess non-response at follow-up, demographic and health characteristics of participants with repeated measurements (*n* = 947) were compared to those with only measurements at baseline (*n* = 787) using chi-squared, Mann–Whitney *U* tests, or independent sample *t* tests.

Sleep and 24-h activity rhythms were checked for outliers, above 4 s.d., were set to 4 s.d. from the mean in the same direction. SOL and depressive symptom scores were log-transformed when used as an outcome. For all covariates, missing values were less than 5%, these were handled using the multiple imputation MICE R package (Buuren & Groothuis-Oudshoorn, 2010). Multiple imputation resulted in five imputed datasets for which the pooled statistics are presented (Rubin, 2004). Based on eight independent exposures, we used a Bonferroni-corrected *p* value = 0.00625, based on an alpha of 0.05, to account for multiple testing. For all estimates, the 95% confidence intervals (CIs) were calculated and presented. Analyses were performed in R version R 3.6.3 (R Foundation for Statistical Computing, Vienna, Austria; http://www.R-project.org).

Cross-sectional linear regression models were used to estimate the association of 24-h activity rhythms and sleep with depressive symptoms. All associations were analyzed using three different models: in model 1 we adjusted for sex and age only. In model 2 we adjusted for above-mentioned confounders, age, sex, cohort, employment, education, partnership, smoking, coffee intake, alcohol consumption, and BMI. In model 3 we additionally adjusted for general cognitive function, which could be a confounder or mediator. For TST and TIB, analyses were repeated using quadratic terms to assess non-linear associations.

Linear-mixed models were used to assess repeated measurements over time. To account for the within person correlation between measurements, a random intercept and slope were included in all linear-mixed models. All linear-mixed models included the baseline value of the determinant and an interaction term of the baseline determinant with time. The effect estimate of the baseline determinant describes how the determinant at baseline is associated with the outcome on average over time. The interaction term between the baseline determinant and time describes how the determinant at baseline is associated with changes in outcome over time. All associations were adjusted according to the above specified models 1, 2, and 3, with all models additionally adjusted for follow-up time and type of actigraphy recorder at follow-up. For TST and TIB, analyses were repeated using quadratic terms to assess non-linear associations.

We repeated all analyses with a recalculated depressive symptoms score, excluding the question assessing problems with sleep (question 11).

## Results

At baseline, 1734 participants were included with a mean age of 62.3 ± 9.3 years and 55% women (see Table 1). Valid repeated measurements were obtained for 947 participants with a median follow-up time of 6 years (IQR = 5.6–6.3). Participants with only baseline measurements were older (*t* = 5.43, *p* < 0.001), more often women (χ^2^ = 8.96, *p* = 0.002), more often without a partner (χ^2^ = 15.17, *p* < 0.001), lower educated (χ^2^ = 17.65, *p* < 0.001), more often unemployed (χ^2^ = 6.11, *p* = 0.050), and overall less healthy compared to participants with repeated measurements (online Supplementary Table S1). Additionally, their 24-h activity rhythm was more disturbed and their sleep poorer (online Supplementary Table S1).

**Table 1.** Baseline characteristics of the study population at baseline (December 2004–April 2007), Rotterdam Study

	*N*	(%)	*M*	s.d.
Participants	1734			
Age (years)			62.3	9.3
Women	951	54.8		
Having a partner	1363	78.7		
Employed	608	35.1		
Education				
Primary	140	8.1		
Low	701	40.8		
Intermediate	519	30.2		
High	360	20.9		
Depressive symptoms (score)^a^^,^^b^			3.0	1–7
Cognitive status (score)^c^			28.0	1.6
BMI (kg/m^2^)			27.8	4.2
Alcohol (cups/day)^a^^,^^d^			0.4	0.0–1.3
Coffee (cups/day)^a^^,^^d^			1.0	0.1–1.7
Smoking				
Non-smoker	531	30.6		
Former smoker	892	51.5		
Current smoker	311	17.9		
IS (score)			0.77	0.12
IV (score)			0.43	0.14
TIB (h:min)			8:12	0:50
TST (h:min)			6:05	0:53
SE (%)			74.5	8.6
SOL (min)			21.0	16.0
WASO (min)			63.0	26.0
Self-rated sleep quality (score)			4.0	3.6

s.d., standard deviation.

Sleep quality is missing for 29 participants (1.7%), partnership for 2 (0.1%), education for 14 (0.8%), alcohol and coffee intake for 36 (2.1%), BMI for 15 (0.9%), and general health score for 66 (3.8%). For other variables there are no missing values.

a Median and IQR.

b Assessed using the Center for Epidemiologic Studies Depression scale.

c Assessed using the Mini-Mental State Exam.

d Alcohol and coffee intake were assessed using the actigraphy sleep dairy.

### Cross-sectional associations

At baseline, a fragmented 24-h activity rhythm (*B* = 0.958, 95% CI = 0.581–1.336), long TIB (*B* = 0.113, 95% CI = 0.052–0.174), low SE (*B* = −0.014, 95% CI = −0.019 to −0.008), long SOL (*B* = 0.009, 95% CI = 0.006–0.012), and low self-rated sleep quality (where a high score reflects poor sleep quality, *B* = 0.116, 95% CI = 0.103–0.129) were associated with more log-transformed depressive symptoms when adjusted for confounders (model 2) (see Table 2). When assessing quadratic association, TST and TIB were not significantly associated with depressive symptoms.

**Table 2.** Cross-sectional associations of 24-h activity rhythms and sleep with depressive symptoms at baseline

	*B*	95% CI	*β*	*p* value
IS	−0.501	−0.932 to −0.069	−0.056	0.023
IV	0.958	0.581–1.336	0.125	<0.001
TIB (h)	0.113	0.052–0.174	0.094	<0.001
TST (h)	−0.043	−0.099–0.014	−0.039	0.14
SE (%)	−0.014	−0.019 to −0.008	−0.116	<0.001
SOL (min)	0.009	0.006–0.012	0.145	<0.001
WASO (min)	0.002	0.000–0.003	0.040	0.09
Self-rated sleep quality score	0.116	0.103–0.129	0.408	<0.001

CI, confidence interval for *B*.

*B* is the effect estimate of the determinant and *β* is the standardized effect estimate of the determinant. Effect estimates were obtained using cross-sectional linear regression models, adjusted for age, sex, cohort, actigraphy device at follow-up, employment, education, partnership, smoking, alcohol consumption, coffee intake, and BMI (model 2). Multiple testing-corrected *p* value = 0.00625.

### Longitudinal associations

Poor sleep at baseline was associated with more depressive symptoms over 6 years of follow-up and slower increase of depressive symptoms over time (Table 3; online Supplementary Fig. S2). Each 1-point higher fragmentation of the 24-h activity rhythm was associated with 1.002 (95% CI = 0.641–1.363) more log-transformed depressive symptoms over 6 years of follow-up when adjusted for confounders (model 2). Similarly, a long TIB (*B* = 0.111, 95% CI = 0.053–0.169), low SE (*B* = −0.015, 95% CI = −0.020 to −0.009), long SOL (*B* = 0.009 (95% CI = 0.006–0.012), and high self-rated sleep quality score (where a high score reflects poor sleep quality, *B* = 0.112, 95% CI = 0.099–0.124) increased depressive symptoms. When assessing quadratic associations, TST and TIB were not significantly associated with depressive symptoms. With every 1-min longer SOL at baseline, the yearly increase of depressive symptoms was reduced with 0.001 (95% CI = −0.001 to 0.000) after adjustment for confounders (model 2). Similarly, with every 1-point higher self-rated sleep quality, the yearly increase of depressive symptoms was reduced with 0.005 (95% CI = −0.008 to −0.002). In other words, long SOL (online Supplementary Fig. S2F) and poor self-rated sleep (online Supplementary Fig. S2H) at baseline were associated with a less steep increase of depressive symptoms over time.

**Table 3.** Longitudinal association of 24-h activity rhythms and sleep with depressive symptoms over time

	*B*	95% CI	*β*	*p* value
IS				
IS	−0.528	−0.943 to −0.114	−0.059	0.012
IS × time	−0.010	−0.103 to 0.084	−0.001	0.84
IV				
IV	1.002	0.641–1.363	0.131	<0.001
IV × time	−0.029	−0.109 to 0.051	−0.004	0.48
TIB				
TIB	0.111	0.053–0.169	0.092	<0.001
TIB × time	−0.007	−0.019 to 0.006	−0.005	0.29
TST				
TST	−0.051	−0.103 to 0.002	−0.046	0.06
TST × time	0.006	−0.005 to 0.017	0.005	0.30
SE				
SE	−0.015	−0.020 to −0.009	−0.126	<0.001
SE × time	0.001	0.000–0.003	0.011	0.030
SOL				
SOL	0.009	0.006–0.012	0.143	<0.001
SOL × time	−0.001	−0.001 to 0.000	−0.014	0.006
WASO				
WASO	0.002	0.000–0.004	0.049	0.034
WASO × time	0.000	0.000–0.001	0.003	0.61
Self-rated sleep quality score				
Sleep quality	0.112	0.099–0.124	0.394	<0.001
Sleep quality × time	−0.005	−0.008 to −0.002	−0.019	<0.001

TST, total sleep time; TIB, time in bed; SE, sleep efficiency; WASO, wake after sleep onset; SOL, sleep onset latency; IS, interdaily stability; IV, intradaily variability; CI, confidence interval for *B*.

*B* is the effect estimate of the determinant and *β* is the standardized effect estimate of the determinant. Effect estimates were obtained using linear-mixed models, adjusted for age, sex, cohort, actigraphy device at follow-up, employment, education, partnership, smoking, alcohol consumption, coffee intake, and BMI (model 2). Estimates should be interpreted as changes in the log-transformed depressive symptoms score. Multiple testing-corrected *p* value = 0.00625.

Vice versa, having more depressive symptoms at baseline was also associated with decreased sleep over 6 years of follow-up and slower worsening of sleep over time (Table 4; online Supplementary Fig. S3). A 1-point higher depressive symptoms score at baseline was associated with a 0.002 (95% CI = 0.001–0.003) increased fragmentation of the 24-h activity rhythm over 6 years of follow-up when adjusted for confounders (model 2). Similarly, having more depressive symptoms was associated with increased TIB (*B* = 0.009, 95% CI = 0.004–0.015), decreased SE (*B* = −0.140, 95% CI = −0.196 to −0.084), increased log-transformed SOL (*B* = 0.013, 95% CI = 0.008–0.018), and decreased self-rated sleep quality score (*B* = 0.193, 95% CI = 0.171–0.215, a high score reflects poor sleep quality). Although TST was not significantly associated with a higher intercept, with every 1-point higher depressive symptom score at baseline the yearly decrease of TST was reduced with approximately 0.002 h (95% CI = 0.001–0.003) after adjustment for confounders (model 2). Similar results were observed for SE (*B* = 0.022, 95% CI = 0.011–0.033) and self-rated sleep quality (*B* = −0.014, 95% CI = −0.019 to −0.009). In other words, more depressive symptoms at baseline were associated with a less steep decrease in TST (online Supplementary Fig. S3D) and a less steep increase in SE (online Supplementary Fig. S3E), and self-rated sleep quality score (online Supplementary Fig. S3H) over time.

**Table 4.** Longitudinal association of depressive symptoms with 24-h activity rhythms and sleep over time

	*B*	95% CI	*β*	*p* value
IS				
IS	−0.001	−0.002 to 0.000	−0.008	0.021
IS × time	0.000	0.000–0.000	−0.001	0.32
IV				
IV	0.002	0.001–0.003	0.016	<0.001
IV × time	0.000	0.000 to −0.000	−0.002	0.025
TIB				
TIB	0.009	0.004–0.015	0.011	0.001
TIB × time	0.000	−0.001 to 0.001	0.000	0.88
TST				
TST	−0.006	−0.012 to 0.000	−0.006	0.06
TST × time	0.002	0.001–0.003	0.002	<0.001
SE				
SE	−0.140	−0.196;−0.084	−0.017	<0.001
SE × time	0.022	0.011;0.033	0.003	<0.001
SOL				
SOL	0.013	0.008;0.018	0.018	<0.001
SOL × time	−0.001	−0.002;0.000	−0.002	0.046
WASO				
WASO	0.161	−0.012;0.334	0.006	0.07
WASO × time	−0.040	−0.072;−0.008	−0.002	0.015
Self-rated sleep quality score				
Sleep quality	0.193	0.171;0.215	0.056	<0.001
Sleep quality × time	−0.014	−0.019;−0.009	−0.004	<0.001

TST, total sleep time; TIB, time in bed; SE, sleep efficiency; WASO, wake after sleep onset; SOL, sleep onset latency; IS, interdaily stability; IV, intradaily variability; CI, confidence interval for *B*.

*B* is the effect estimate of the determinant and *β* is the standardized effect estimate of the determinant. Effect estimates were obtained using linear-mixed models, adjusted for age, sex, cohort, actigraphy device at follow-up, employment, education, partnership, smoking, alcohol consumption, coffee intake, and BMI (model 2). Estimates should be interpreted as changes in the log-transformed depressive symptoms score. Multiple testing-corrected *p* value = 0.00625.

Analyses adjusted for sex and age only showed similar results (data not shown). Results did not change substantially when estimates were additionally adjusted for general cognitive function (model 3, online Supplementary Tables S2 and S3).

### Sensitivity analyses

Results did not substantially change when analyses were repeated using the depressive symptoms score without the sleep item (data not shown).

## Discussion

We demonstrated a bidirectional association of 24-h activity rhythms and sleep with depressive symptoms in a population-based cohort of middle-aged and elderly persons. For 24-h activity rhythms, only fragmentation of the 24-h activity rhythm was bidirectionally associated with more depressive symptoms. For sleep, we observed that long TIB, low actigraphy-estimated SE, long actigraphy-estimated SOL, and poor self-rated sleep quality were bidirectionally associated with more depressive symptoms.

Within a single-study population, we demonstrated a temporal association of fragmented 24-h activity rhythms and sleep with depressive symptoms over time, and vice versa, indicating a bidirectional association. This is in line with previous studies which have showed either direction (Fang et al., 2019; Maglione et al., 2014), but never assessed both directions using objectively estimated sleep measures in a single-study population (Maglione et al., 2014; Smagula et al., 2017). A bidirectional association of 24-h activity rhythms and sleep with depressive symptoms seems plausible. On the one hand, fragmented 24-h activity rhythms and sleep disturbances may cause depressive symptoms through internal desynchronization of 24-h rhythms (Pandi-Perumal et al., 2009), impaired Rapid Eye Movement (REM) sleep (Wang et al., 2015), or impaired cognitive function (Helfrich et al., 2019; Muzur, Pace-Schott, & Hobson, 2002) for example. On the other hand, depressed people often experience more stress and worry, potentially linked to a moor pore function of the pre-frontal cortex (Belleau, Treadway, & Pizzagalli, 2019; Liu et al., 2017), which could also lead to difficulties in falling asleep, lower SE, and poorer self-rated sleep quality (Hall et al., 2015; Van Laethem, Beckers, van Hooff, Dijksterhuis, & Geurts, 2016). In addition, depressed people may experience difficulties in performing daily tasks for a long period of time (Liu, Peng, Zhang, & Tang, 2018), which could lead to less intensive activity peaks and more fragmentation of 24-h activity rhythms. Of note, within the sleep–depression link, cognitive function is often deemed particularly relevant, as both concepts have direct relations with cognition (Beck, 2013; Belleau et al., 2019; Helfrich et al., 2019). Yet, in our study cognitive function at baseline did not affect the associations of sleep with depression or vice versa. It might be that our general indicator of cognitive function was not sufficiently able to pick up subtle changes in cognition, or that instead of general cognition, one particular construct such as executive function affects the association of sleep and depressive symptoms.

The observed bidirectional association between poor sleep and depressive symptoms could also be explained by a common cause, a third factor that explains changes in the 24-h activity rhythm and sleep as well as depressive symptoms. One such common cause could be degeneration of the SCN, the biological clock of the brain, which is an important cause of increased fragmentation of the 24-h activity rhythm and other sleep problems (Swaab et al., 1985) and is associated with depressive disorders (Costa, Carvalho, & Fernandes, 2013; Vadnie & McClung, 2017). Degeneration of the SCN hampers functioning of structures that affect both sleep and mood, such as the HPA-axis (Asarnow, 2019; Murri et al., 2014). The HPA-axis is associated with both sleep disturbances and mood disorders via elevation of cortisol levels and hyperarousal (Bao et al., 2017). Of note, degeneration of the SCN could also reflect associated degeneration in other parts of the brain such as the diencephalon, telencephalon, which includes the previously described prefrontal cortex, and the reticular formation in the brain stem (Mitrushina & Tomaszewski, 2019; Musiek et al., 2018). This degeneration in other parts of the brain might lead to altered cognitive functioning or health perception which might in turn affect both sleep and depression (Leng, Musiek, Hu, Cappuccio, & Yaffe, 2019; Mitrushina & Tomaszewski, 2019; Musiek et al., 2018).

Our results furthermore demonstrate that poor sleep at baseline is associated with a slower increase of depressive symptoms. Vice versa, having more depressive symptoms at baseline was also associated with a slower worsening of sleep. As we only have measured sleep and depression at two time points it is difficult to explain these somewhat counterintuitive findings. It could be hypothesized that persons with poor sleep at baseline also have more depressive symptoms at baseline and therefore have less possibility for an increase of depressive symptoms or worsening of sleep. Yet, this seems unlikely in our population-based sample which shows lower scores on sleep quality and depression than clinical samples. In addition, people with poor sleep at baseline still seem to have stable high depressive symptoms over time. Similarly, people with more depressive symptoms at baseline seem to have stable poor sleep over time.

Commonly, a discrepancy between objective and self-rated measures is observed for the association of sleep with depression (Van Den Berg et al., 2008), where self-rated measures reflect more of the subjective experience and tend to be more strongly related to depressive symptoms (Maglione et al., 2014; Mitrushina & Tomaszewski, 2019). Dysfunction of the reticular activating system, important for a person's consciousness and health perception (Brudzynski, 2014; Hudson, 2009), could potentially lead to a heightened consciousness about their sleep and depressive symptoms. Based on our standardized results it seems that subjective, or self-rated, sleep indeed shows stronger temporal associations for sleep and depressive symptoms than purely objectively estimated measures such as sleep duration and sleep fragmentation. Additionally, aspects of sleep that integrate information from the sleep diary, which is self-reported, such as SOL and TIB, also seem to be more strongly associated with depressive symptoms.

Several limitations need to be taken into account. First, we used two different actigraphy devices at follow-up. Although we have controlled for this in our analyses this might have induced measurement error. Second, our nonresponse analyses revealed differences between participants with repeated measurements and participants with only a baseline measurement, possibly introducing selection bias. Finally, although having repeated measurements is a strength, we were only able to include two time points, 5 to 9 years apart. We could therefore not take into account fluctuations in depressive symptoms and sleep occurring between these two time points. Third, the number of reported depressive symptoms is relatively low in our sample, about 9% of our study population reached the cut-off for clinically relevant depressive symptoms, which fits with the population-based design. Our approach however does allow us to take into account subclinical symptoms of depression which are more frequent at older ages. Finally, the effect sizes in our study are relatively small, in part as a consequence of the population-based setting, which might limit the clinical relevance. Nevertheless, having repeated objective sleep measurements and repeated depressive symptom assessments over this period of time is unique in this field and allowed us to assess bidirectionality within one study population.

In our study population of middle-aged and elderly we demonstrated a bidirectional association between fragmented 24-h activity rhythms, low actigraphy-estimated SE, long actigraphy-estimated SOL, and poor self-rated sleep quality and depressive symptoms over time. This bidirectionality in both objectively estimated aspects of sleep as well as self-rated sleep quality with depressive symptoms over time should be taken into account when assessing underlying mechanisms and when developing and improving non-pharmacological and pharmacological prevention strategies and treatments for poor sleep and depression.

## Supplementary material

For supplementary material accompanying this paper visit https://doi.org/10.1017/S003329172100297X. click here to view supplementary material
