# Supplementary material for: The bidirectional association of 24-h activity rhythms and sleep with depressive symptoms in middle-aged and elderly persons
Source: Psychol Med. 2021 Aug 11;53(4):1418–25. doi: 10.1017/S003329172100297X (PMC10009400; doi:10.1017/S003329172100297X)
Supplement: Supplementary file 1 [file S003329172100297Xsup.zip › S003329172100297Xsup002.docx]

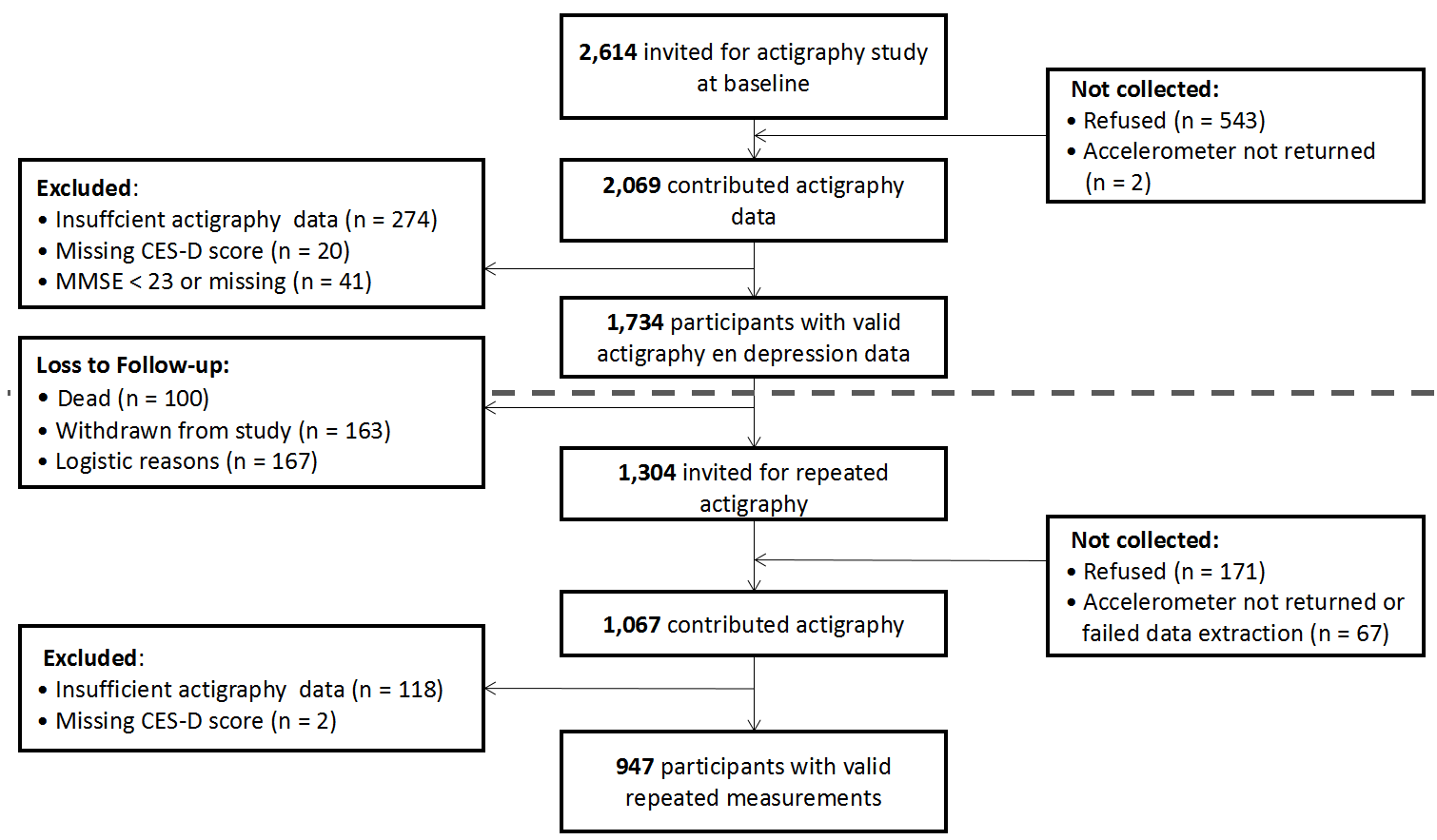


**Supplementary Figure 1**

*Flow diagram of the study population . Valid baseline data for actigraphy and depressive symptoms was collected for 1,734 participants between 2006 and 2008. Of those, valid repeated assessment Valid baseline data for actigraphy and depressive symptoms was performed for 947 participants between 2009 and 2012..Abbreviations; CES-D: Center for Epidemiological Depression scale; MMSE: Mini-Mental State Exam.*


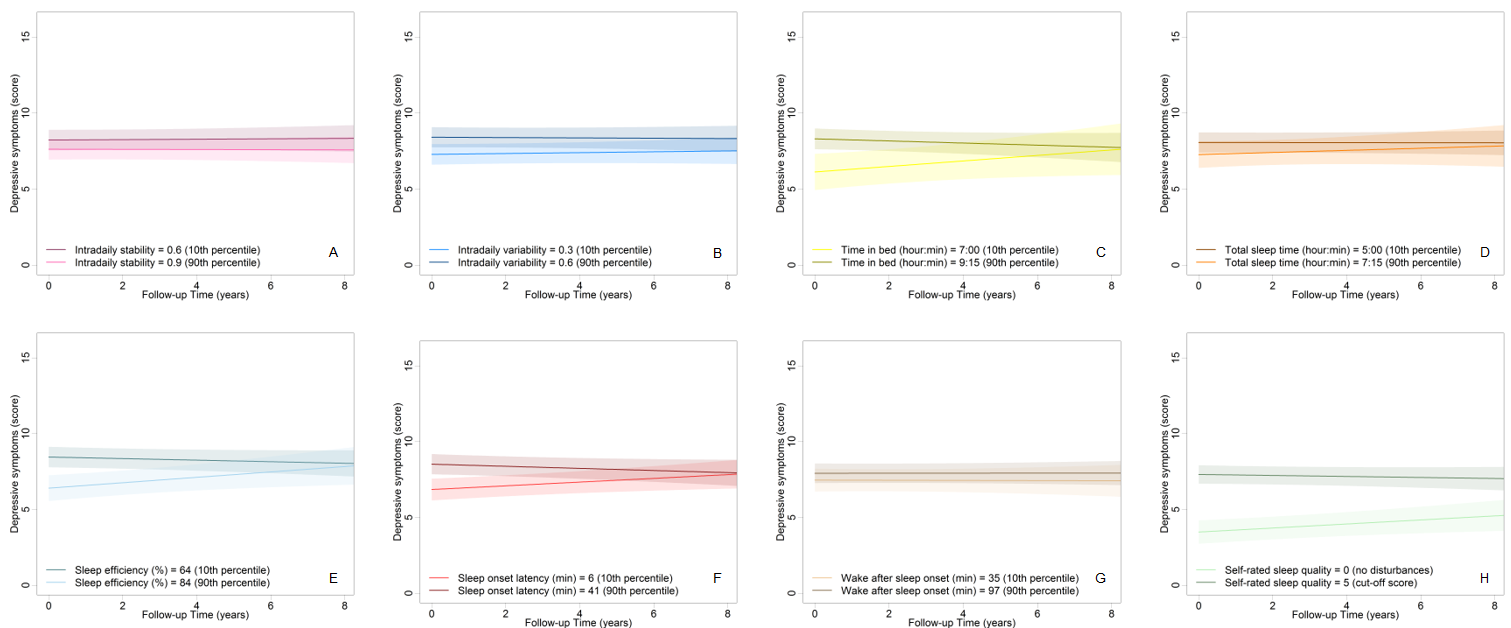


**Supplementary Figure 2**

*Sleep and 24-hour activity rhythms predicting depressive symptoms across time. Each plot depicts how depressive symptoms would develop over time for an average person scoring the 10^th^ or 90^th^ percentile for Interdaily Stability (A), Intradaily Variability (B), Time In Bed (C), Total Sleep Time (D), Sleep Efficiency (E), Sleep Onset Latency (F), and Wake After Sleep Onset (G). Darker colors indicate poor sleep, lighter colors indicate good sleep. For self-rated sleep quality (H) a line is plotted for the average person with no self-rated sleep disturbances (light green) and an average person with poor self-rated sleep quality (cut-off score, dark green).*


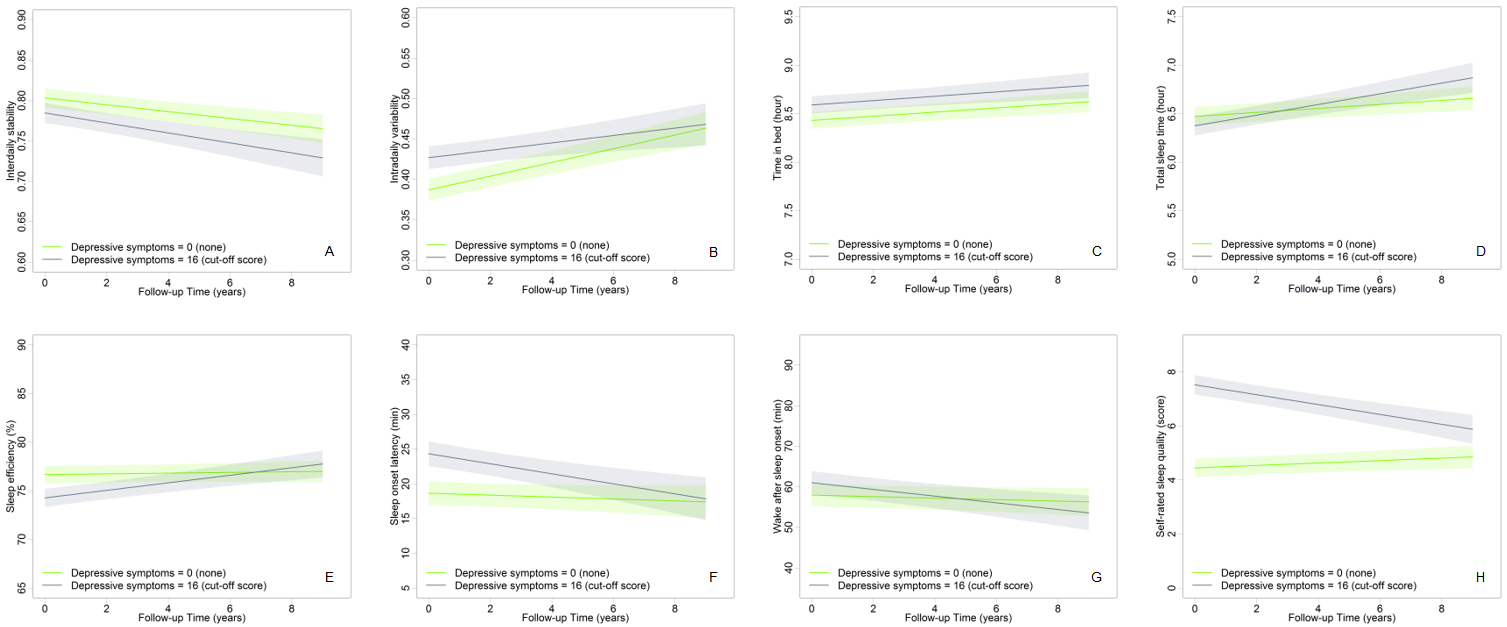


**Supplementary Figure 3**

*Depressive symptoms predicting actigraphy-estimated and self-rated sleep time. Each plot depicts how Interdaily Stability (A), Intradaily Variability (B), Time In Bed (C), Total Sleep Time (D) Sleep Efficiency (E), Sleep Onset Latency (F), Wake After Sleep Onset (G), and self-rated sleep quality (H) would develop over time for the average person without depressive symptoms (blue ) or with clinically relevant depressive symptoms (green*).
