# Supplementary material for: The bidirectional association of 24-h activity rhythms and sleep with depressive symptoms in middle-aged and elderly persons
Source: Psychol Med. 2021 Aug 11;53(4):1418–25. doi: 10.1017/S003329172100297X (PMC10009400; doi:10.1017/S003329172100297X)
Supplement: Supplementary file 1 [file S003329172100297Xsup.zip › S003329172100297Xsup003.docx]

**Supplementary Table 1**

*Baseline characteristics of the study population at baseline (December 2004 – April 2007), Rotterdam study. Comparison of participants with baseline measures only to participants with repeated measures.*

|  | | Baseline | Repeated | Test-statistic | p-value |
| --- | --- | --- | --- | --- | --- |
| Participants | | 787 | 947 |  |  |
| Age (years) | | 63.6 ± 10.7 | 61.1 ± 7.6 | t = 5.43 | p < 0.001 |
| Women | | 463 (59 %) | 488 (52 %) | *χ*^2^ = 8.96 | p = 0.003 |
| Having a partner | | 585 (74 %) | 778 (82 %) | *χ*^2^ = 15.17 | p < 0.001 |
| Employed | | 251 (32 %) | 357 (38 %) | *χ*^2^ = 6.11 | p = 0.013 |
| Education | |  |  | *χ*^2^ = 17.65 | p < 0.001 |
| Primary | | 79 (10 %) | 61 (6 %) |  |  |
| Low | | 335 (43 %) | 366 (39 %) |  |  |
| Intermediate | | 232 (30 %) | 287 (30 %) |  |  |
| High | | 135 (17 %) | 225 (24 %) |  |  |
| Depressive symptoms (score) ^a, b^ | | 3.0 (1 – 8) | 3.0 (0 – 7) | U = 406150 | p < 0.001 |
| Cognitive status (score) ^c^ | | 27.9 ± 1.7 | 28.2 ± 1.6 | U = 328480, | p < 0.001 |
| Body mass index (kg/m^2^) | | 27.9 ± 4.3 | 27.8 ± 4.2 | t = 0.39 | p = 0.70 |
| Alcohol(cups/day) ^a, c^ | | 0.3 (0 – 1.1) | 0.5 (0 – 1.3) | U = 327420 | p = 0.004 |
| Coffee(cups/day) ^a, c^ | | 0.9 (0.1 – 1.4) | 1 (0.3 – 1.8) | U = 314260 | p < 0.001 |
| Smoking |  |  |  | *χ*^2^ = 10.56 | p = 0.005 |
| Non smoker | | 231 (29 %) | 300 (32 %) |  |  |
| Former smoker | | 398 (50 %) | 503 (53 %) |  |  |
| Current smoker | | 167 (21 %) | 144 (15 %) |  |  |
| Interdaily stability (score) | | 0.77 ± 0.12 | 0.78 ± 0.11 | t = -2.75 | p = 0.006 |
| Intradaily variability (score) | | 0.44 ± 0.14 | 0.41 ± 0.12 | t = 4.12 | p < 0.001 |
| Time in bed (hours) | | 8:17 ± 0:50 | 8:07 ± 0:50 | t = 4.5 | p < 0.001 |
| Total sleep time (hours) | | 6:10 ± 0:54 | 6:04 ± 0:54 | t = 1.94 | p = 0.045 |
| Sleep efficiency (%) | | 74.0 (8.5) | 74.9 ± 8.4 | t = -2.11 | p = 0.036 |
| Sleep onset latency (min) | | 22 ± 16 | 20 ± 16 | t = 2.24 | p = 0.018 |
| Wake after sleep onset (min) | | 64 ± 27 | 61 ± 25 | t = 2.38 | p = 0.025 |
| Self-rated sleep quality (score) | | 4.2 ± 3.7 | 3.8 ± 3.4 | t = 2.12 | p = 0.034 |

**Abbreviations:** BMI, body mass index; ADL , Activities of Daily Living; MMSE, Mini-Mental State Exam; CI, Confidence Interval.

^a^ Median and Interquartile Range.
^b^ Assessed using the Center for Epidemiologic Studies Depression scale. ^c^ Assessed using the Mini-Mental State Exam.

^d^ Alcohol and coffee intake were assessed using the actigraphy sleep dairy.

**Supplementary Table 2**

*Longitudinal association of 24-hour activity rhythms and sleep with depressive symptoms over time, additionally adjusted for general cognitive function*

|  | B | 95%CI | β | p-value |  |
| --- | --- | --- | --- | --- | --- |
| Interdaily stability |  |  |  |  |  |
| IS | -0.501 | (-0.914;-0.088) | -0.057 | 0.018 |  |
| IS*time | -0.010 | (-0.104;0.083) | 0.015 | 0.83 |  |
| Intradaily variability |  |  |  |  |  |
| IV | 0.945 | (0.583;1.307) | 0.119 | <0.001 |  |
| IV*time | -0.029 | (-0.109;0.051) | -0.007 | 0.48 |  |
| Time in bed |  |  |  |  |  |
| TIB | 0.102 | (0.043;0.160) | 0.074 | 0.001 |  |
| TIB*time | -0.006 | (-0.019;0.006) | -0.005 | 0.867 |  |
| Total sleep time |  |  |  |  |  |
| TST | -0.053 | (-0.105;0.000) | -0.038 | 0.048 |  |
| TST*time | 0.006 | (-0.005;0.017) | -0.028 | 0.29 |  |
| Sleep efficiency |  |  |  |  |  |
| SE | -0.014 | (-0.020;-0.009) | -0.102 | <0.001 |  |
| SE*time | 0.001 | (0.000;0.003) | -0.018 | 0.030 |  |
| Sleep onset latency |  |  |  |  |  |
| SOL | 0.008 | (0.006;0.011) | 0.111 | <0.001 |  |
| SOL*time | -0.001 | (-0.001;0.000) | -0.014 | 0.008 |  |
| Wake after sleep onset | |  |  |  |  |
| WASO | 0.002 | (0.000;0.004) | 0.052 | 0.042 |  |
| WASO*time | 0.000 | (0.000;0.001) | -0.014 | 0.63 |  |
| Self-rated sleep quality score | |  |  |  |  |
| Sleep quality | 0.112 | (0.100;0.124) | 0.363 | <0.001 |  |
| Sleep quality*time | -0.005 | (-0.008;-0.002) | -0.020 | <0.001 |  |

Abbreviations: TST, Total Sleep Time; TIB, Time In Bed; SE, Sleep Efficiency; WASO, Wake After Sleep Onset; SOL, Sleep Onset Latency; IS, Interdaily stability; IV, Intradaily variability; CI, Confidence Interval for B. B is the effect estimate of the determinant, β the standardized effect estimate of the determinant. Effect estimates were obtained using linear mixed models, adjusted for age, sex, cohort, actigraphy device at follow-up, employment, education, partnership, smoking, alcohol consumption, coffee intake, body mass index, and general cognitive function (model 3). Estimates should be interpreted as changes on the log-transformed depressive symptoms score. Multiple testing corrected p-value = 0.00625.

**Supplementary Table 3**

*Longitudinal association of depressive symptoms with 24-hour activity rhythms and sleep over time, additionally adjusted for general cognitive function*

|  | B | 95%CI | β | p-value |  |
| --- | --- | --- | --- | --- | --- |
| Interdaily stability |  |  |  |  |  |
| IS | -0.001 | (-0.002;0.000) | -0.009 | 0.032 |  |
| IS*time | 0.000 | (0.000;0.000) | 0.006 | 0.31 |  |
| Intradaily variability |  |  |  |  |  |
| IV | 0.002 | (0.001;0.003) | 0.011 | <0.001 |  |
| IV*time | 0.000 | (0.000;0.000) | 0.010 | 0.026 |  |
| Time in bed |  |  |  |  |  |
| TIB | 0.009 | (0.043;0.160) | 0.010 | 0.002 |  |
| TIB*time | 0.000 | (-0.001;0.001) | 0.004 | 0.871 |  |
| Total sleep time |  |  |  |  |  |
| TST | -0.006 | (-0.012;0.000) | -0.002 | 0.06 |  |
| TST*time | 0.002 | (0.001;0.003) | 0.007 | <0.001 |  |
| Sleep efficiency |  |  |  |  |  |
| SE | -0.135 | (-0.191;-0.078) | -0.011 | <0.001 |  |
| SE*time | 0.022 | (0.011;0.033) | 0.005 | <0.001 |  |
| Sleep onset latency |  |  |  |  |  |
| SOL | 0.012 | (0.007;0.017) | 0.014 | <0.001 |  |
| SOL*time | -0.001 | (-0.002;0.000) | -0.004 | 0.049 |  |
| Wake after sleep onset | |  |  |  |  |
| WASO | 0.152 | (-0.022;0.325) | 0.003 | 0.09 |  |
| WASO*time | -0.040 | (-0.072;-0.008) | 0.003 | 0.015 |  |
| Self-rated sleep quality score | |  |  |  |  |
| Sleep quality | 0.196 | (0.173;0.218) | 0.049 | <0.001 |  |
| Sleep quality*time | -0.014 | (-0.019;-0.010) | -0.003 | <0.001 |  |

Abbreviations: TST, Total Sleep Time; TIB, Time In Bed; SE, Sleep Efficiency; WASO, Wake After Sleep Onset; SOL, Sleep Onset Latency; IS, Interdaily stability; IV, Intradaily variability; CI, Confidence Interval for B. B is the effect estimate of the determinant, β the standardized effect estimate of the determinant. Effect estimates were obtained using linear mixed models, adjusted for age, sex, cohort, actigraphy device at follow-up, employment, education, partnership, smoking, alcohol consumption, coffee intake, body mass index, and general cognitive function (model 3). should be interpreted as changes on the log-transformed depressive symptoms score. Multiple testing corrected p-value = 0.00625.
